# Harnessing coupled nanolasers near exceptional points for directional emission

**DOI:** 10.1126/sciadv.adr8283

**Published:** 2024-11-08

**Authors:** Guilhem Madiot, Quentin Chateiller, Alexandre Bazin, Patricia Loren, Konstantinos Pantzas, Grégoire Beaudoin, Isabelle Sagnes, Fabrice Raineri

**Affiliations:** ^1^ Université Côte d’Azur, CNRS, Institut de Physique de Nice, 06200, Nice, France.; ^2^ Centre de Nanosciences et de Nanotechnologies, CNRS, Université Paris-Saclay, 10 Bd Thomas Gobert, 91120 Palaiseau, France.

## Abstract

Tailoring the losses of optical systems within the frame of non-Hermitian physics has appeared very fruitful in the past few years. In particular, the description of exceptional points (EPs) with coupled resonators has become widespread. The on-chip realization of these functionalities is crucial for integrated nanophotonics but requires fine control techniques of the nanodevice properties. Here, we demonstrate pump-controlled directional emission of two coupled nanolasers that distantly interact via an integrated waveguide. This coupling scheme unusually enables both frequency and loss couplings between two cavities, which can be advantageously exploited to reach EPs by either detuning the cavities or controlling the gain of nanolasers. The system can be readily reconfigured from bidirectional to unidirectional emission by adjusting the pump power.

## Teaser

Controllable directionality in the emission of coupled nanolasers connected by a waveguide is shown.

## INTRODUCTION

Exceptional points (EPs) are degeneracies in the complex eigenspectrum of coupled resonators where at least two eigenvalues coalesce, with their respective eigenvectors (*1*–*3*). Their presence is associated to a rich phenomenology including spatial (*4*–*6*) or spectral (*7*, *8*) nonreciprocity, topological singularities (*9*–*12*), or enhanced sensitivity (*13*–*17*). In practice, to reach an EP, the coupling rate must be exactly compensated by an asymmetry in the resonators’ natural properties, i.e., frequencies and loss rates. Hence, this operation requires a fine control over the former and/or over the coupling constant itself. Most demonstrations exploit an evanescent coupling between two resonators, leading to a pure energy repulsion, i.e., a real splitting between the resulting quasi-normal modes (QNMs). In this case, an EP can only be reached by introducing an imbalance between the resonators’ dissipation rates. Meanwhile, their frequencies must be perfectly tuned. This situation defines the well-known “gain-loss” configuration, where gain is anti-symmetrically distributed within two resonators with identical natural frequencies, enabling parity-time (PT) symmetry to be broken or restored by crossing an EP (*18*–*20*).

EPs can also be reached if the coupling has a dissipative contribution, i.e., if the coupling constant is complex in the non-Hermitian physics formalism. Few examples include, e.g., multimode cavity optomechanics, where the light-mediated effective coupling between two micromechanical resonators leads to both frequency and loss splitting in the eigenspectrum (*8*, *21*–*23*). Several works have shown that a loss splitting can be obtained in microring resonators, by coupling the clockwise and counter clockwise modes via a scattering mechanism (*4*, *5*, *24*).

**Fig. 1.**
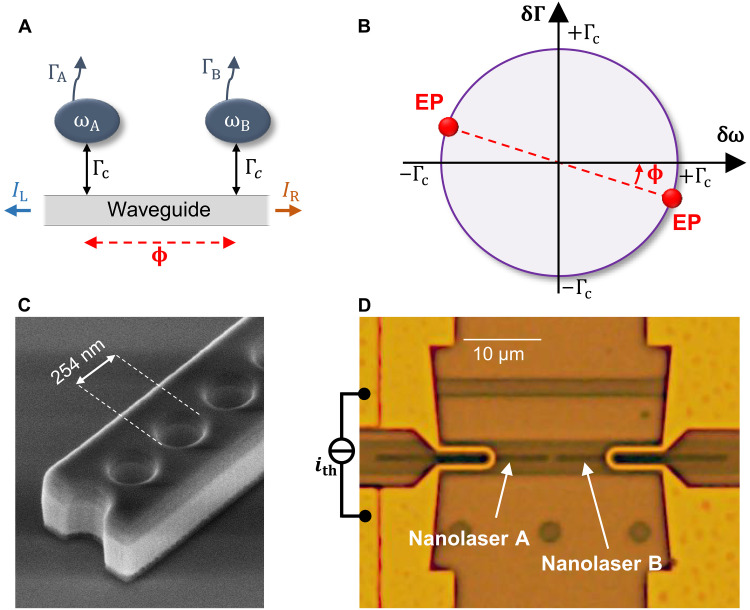
EPs with waveguide-coupled cavities. (**A**) Two waveguide-coupled nanocavities with independent loss rates and frequencies. (**B**) Localization of the EPs in the parameter space {δω, δΓ}, for a given coupling phase ϕ. (**C**) Scanning electron microscopy micrograph of an InP photonic crystal nanolaser. (**D**) Microscope image of the coupled nanolasers with integrated thermoresistive gold nanowires used to tune the nanolasers frequencies.

Loss splitting has also been recognized for a long time as an intriguing consequence of waveguide-coupled standing-wave cavities as described by coupled mode theory (*25*–*27*). The coupling rate between two cavities interacting via a waveguide, as depicted in Fig. 1A, takes the form *K* = Γ_c_*e*^*i*ϕ^. Here, Γ_c_ is the cavity-to-waveguide coupling rate, and ϕ is the phase acquired by the light between the cavities, referred to as the coupling phase in the following. Assuming an independent cavity decay rates, Γ_A_ and Γ_B_, and natural angular frequencies, ω_A_ and ω_B_, the coupled-mode analysis of this configuration provides the rate equations for the cavity amplitudes, $A\equiv {\left(a_{A}\;a_{B}\right)}^{T}$

$$i\frac{dA}{\mathrm{dt}}=\left(\begin{array}{cc} \omega_{A}-i\Gamma_{A} & -iK\\ -iK & \omega_{B}-i\Gamma_{B} \end{array}\right)A$$ (1)

In the general case, the properties of the QNMs in this system are given by the complex eigenvalues of the above effective Hamiltonian. They involve the cavities’ average properties $\bar{p}=\frac{1}{2}\left(p_{A}+p_{B}\right)$ and dissimilarities, $\delta p=\frac{1}{2}\left(p_{A}-p_{B}\right)$

$$\Lambda_{\pm }=\bar{\omega }-i\bar{\Gamma }\pm \sqrt{{\left(\delta \omega -i\delta \Gamma \right)}^{2}-K^{2}}$$ (2)

By setting the complex splitting to zero in Eq. 2, two EPs are found at positions {δω_EP_ = ±Γ_c_ cos ϕ, δΓ_EP_ = ∓Γ_c_ sin ϕ} of the parameter space, as illustrated in Fig. 1B. It must be noted that the effective Hamiltonian in Eq. 1 is generally not PT symmetric (*9*, *10*), unless the cavities are set in the gain-loss configuration mentioned above, i.e., displaying EPs when ω_A_ = ω_B_, Γ_A_ = −Γ_B_ = Γ_c_, and with $\phi =\frac{\pi }{2}\;\left(\text{mod}\;\pi \right)$. In the proposed modal configuration, the system can be markedly reconfigured by a simple modification of the inter-cavity distance. For example, setting the phase shift to a multiple of π enables the EPs to be reached by varying the frequency detuning rather than the gain.

## RESULTS

To implement this scheme, we use a pair of waveguide-coupled nanolasers. Each laser is formed by an indium-phosphide photonic-crystal nanobeam cavity (*28*) and is integrated over a silicon waveguide (*9*). A scanning electron microscopy image of a single nanolaser is shown in Fig. 1C. The cavity-to-waveguide coupling strength, Γ_c_, is determined by both the waveguide geometry and the cavity-to-waveguide gap (*29*). Meanwhile, the coupling phase ϕ is varied by finely increasing the separation distance between the nanolasers. On the basis of numerical evaluation of the waveguide effective index, *n*_eff_ ≈ 2.3, we fabricate a chip where the coupling distance is incremented by 35 nm. This is equivalent to an increment of the coupling phase by ~π/10. Therefore, we are capable of exploring different coupling regime from purely dispersive to purely dissipative coupling.

Each nanolaser is optically pumped at room temperature by focusing a continuous wave 1064-nm laser diode at its center. Both ends of the underneath waveguide are terminated by a grating coupler and are addressed with two optical fibers to collect the emission and perform a spectral analysis. To precisely control the cavity detuning δω, a gold resistive nanowire is fabricated 1 μm above each nanolaser with SiO_2_ in between, as shown in Fig. 1D. When current flows, the nanowire heats up, causing a redshift of the cavity mode δ_th_ = β_th_*P*_th_ up to 5 nm, where *P*_th_ is the heating electrical power.

**Fig. 2.**
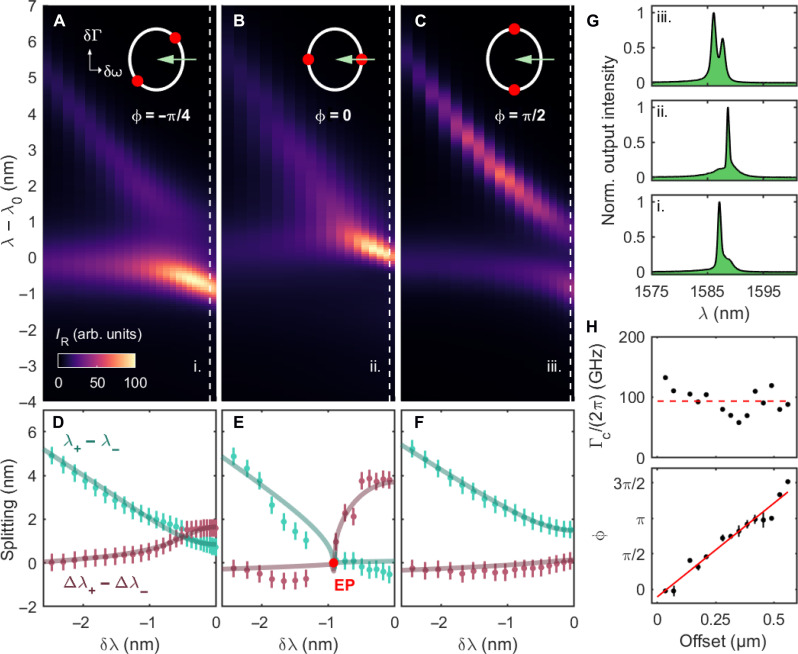
Phase-dependent complex coupling. (**A** to **C**) Experimental emission spectra as a function of the natural cavity detuning δλ for different cavity separation lengths. In white, we represent the parameter space {δω, δΓ} as described in Fig. 1B and illustrate the trajectory performed experimentally (green arrow) as well as the positions of the EPs (red dots). (**D** to **F**) Eigenvalues obtained by fitting the upper data. We show the real (green) and imaginary (red) parts of the complex splitting and fit the data using Eq. 2. (E) An EP is evidenced (red dot) when λ_+_ = λ_−_ and Δλ_+_ = Δλ_−_ are simultaneously verified. (**G**) From top to bottom: Representative spectra are extracted from (A), (B), and (C) (at position indicated by dashed lines) to illustrate purely dispersive, purely dissipative, or complex splitting, respectively. (**H**) The coupling rates Γ_c_ and the coupling phases ϕ are obtained from the fitting in (D), and shown as a function of the cavity separation offset. The rates Γ_c_ distribute around a mean value Γ_c_/(2π) ≈ 93 ± 22 GHz (red dashed line). The values of ϕ are shown with a linear fit (red full line).

Applying identical pumping powers on both cavities, we measure the output spectrum as a function of the heating current, which is directly converted into a cavity detuning, δλ, in Fig. 2 (A to C). The measurement is carried out for three different cavity separation offsets: 315 nm (A), 420 nm (B), and 525 nm (C). In the case (A), we observe a splitting of the QNMs, with both dispersive and dissipative components. We do observe not only an energy repulsion but also an asymmetry in their linewidths, such that one QNM becomes narrower and more intense, whereas the other one broadens. This is verified by fitting the spectrum with a double-voigt function. In Fig. 2D, we show the real part (green) and the imaginary part (red) of the splitting, i.e., λ_+_−λ_−_ and Δλ_+_−Δλ_−_, respectively, with the error bars given by the spectrometer resolution. Fitting the data applying Eq. 2 (full lines), we extract, among others, the cavity-to-waveguide coupling rate Γ_c_, and the coupling phase ϕ. At δλ = 0, we show that the splitting is complex, i.e., both the QNMs spectral positions and linewidths are different. A typical spectrum illustrates this situation in Fig. 2G (top). In the second case (B), we observe a purely dissipative splitting, i.e., one mode broadens, while the other gets narrower as approaching δλ = 0. The eigenvalue analysis and fitting shows that, in this measurement, an EP is crossed within the experimental uncertainties, as pointed out by the red dot in Fig. 2E. Crossing this point is associated with a transition from purely real to purely imaginary splitting. Last, in the third case, the coupling is fully dispersive, i.e., purely real, translating into the typical avoided crossing of the QNMs in the spectrum, whereas their linewidths are relatively identical (Δλ_+_ ≈ Δλ_−_). To reach an EP with this structure, one would have to tune the nanolaser frequencies (δω = 0) and ramp the loss rate difference δΓ. It could be done here by pumping a single nanolaser while keeping the other in an absorption regime (*30*).

The eigenvalue analysis is repeated for different cavity distance offsets, and we evaluate the mean value of the cavity-to-waveguide coupling rate, Γ_c_/(2π) ≈ 93 ± 22 GHz (see Fig. 2H, top). We verify that the phase is proportional to this distance (bottom), where the linear fit provides the effective index of the silicon waveguide, *n*_eff_ ≈ 2.1 ± 0.5, proving consistent with the expectations as mentioned above.

Directional propagation and emission of light constitute one of the most promising phenomena to be exploited with photonic devices set in the vicinity of EPs (*22*, *31*–*33*). In our system, the emission directionality results from the dephasing ϕ between the waves that couples out from the two cavities, into the waveguide. This dephasing allows interferences that can be destructive on the left side but constructive on the right side and vice versa

$$s_{L,R}=i\sqrt{\Gamma_{c}}\;\left(a_{A,B}+e^{i\phi }a_{B,A}\right)$$ (3)

where the left (L) and right (R) output wave amplitudes are defined such that the corresponding intensities write ${I_{L,R}=s_{L,R}}^{2}$.

In a second experiment, we focus on the directional emission of a pair of interacting nanolasers with the measured coupling parameters ϕ ≈ 1.08π and Γ_c_/(2π) = 22.7 GHz. We also determine their respective laser threshold powers independently, *P*_A,*t*_ = 2.2 mW and *P*_B,*t*_ = 4.3 mW, as well as the power dependency of the lasers’ frequencies and damping rates. This dependency occurs through the depletion of carriers in the semiconductor under pumping. Last, we calibrate the heating coefficient βth = 1.94 nm/μW, based on which we set the current *i*_*th*_ such that the nanolasers have identical natural frequencies. We scan *P*_A_ although *P*_B_ is ramped progressively, while the output fields are collected on each termination of the waveguide, and detected by two photoreceivers whose output voltages are read with an oscilloscope.

**Fig. 3.**
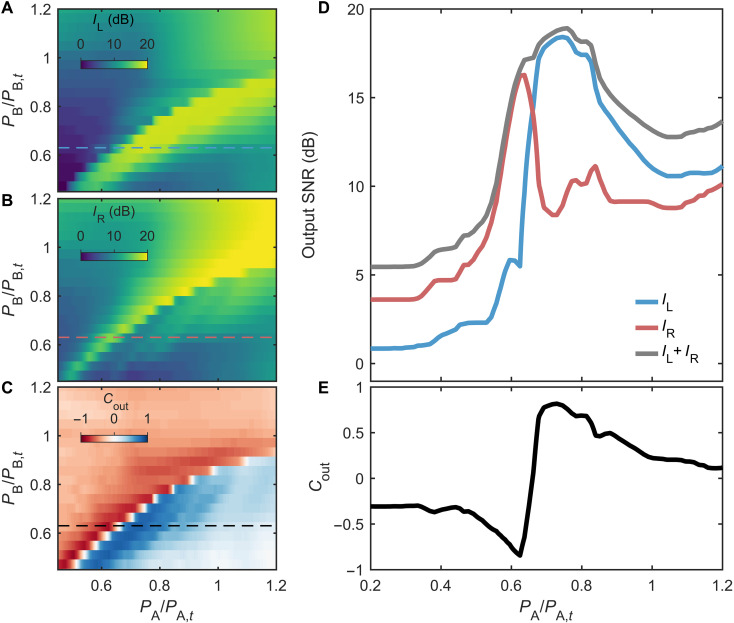
Pump-control of the output emission. (**A**) Output intensity *I*_L_, measured at the left output. (**B**) *I*_R_, measured at the right output. (**C**) Associated output contrast *C*_out_. (**D**) Example measurement of *I*_L_ (blue), *I*_R_ (orange), and *I*_tot_, measured at *P*_B_/*P*_B,*t*_ = 0.5. SNR, signal-to-noise ratio. (**E**) Associated output contrast showing an abrupt transition from negative values (light emission toward the right output port) to positive values (emission toward the left output port).

In Fig. 3 (A and B), we compare the left-side emission, *I*_L_ (A) with the right-side emission, *I*_R_ (B). The intensities are shown as a function of the normalized pump powers, below threshold (*P*_A,B_/*P*_A,B,*t*_ < 1). On both sides, we note a strong enhancement when the normalized powers are equal due to the system symmetry in this situation, i.e., δω = δΓ = 0, leading to a strong loss splitting between the QNMs. Therefore, the enhancement of the emission occurs when the one collective mode whose losses are sufficiently reduced to be overcome by gain, such that it passes the lasing threshold. Although the second QNM’s linewidth has broadened and does not significantly contribute to the emission. More precisely, the loss splitting affects the external losses of the QNMs, such that one mode overcouples to the waveguide, while the other becomes nearly dark, i.e., isolated from the guide. The former QNM can laser only if the pumping rate compensates at least the internal losses of the cavity, which is the case in this experiment.

When comparing the left and right sides, it appears that the relative intensities vary quite significantly along the map. Therefore, it is useful to introduce the output intensity contrast *C*_out_ = (*I*_L_ − *I*_R_)/(*I*_L_ + *I*_R_), as a figure of merit of the emission directionality. It is represented in Fig. 3C, where blue and red colors correspond to a dominant emission toward the left side (*C*_out_ > 0) and toward the right side (*C*_out_ < 0), respectively. The balanced bidirectional emission corresponds to white color (*C*_out_ = 0). This map shows an abrupt variation in the output contrast, mostly when the intensity enhances.

**Fig. 4.**
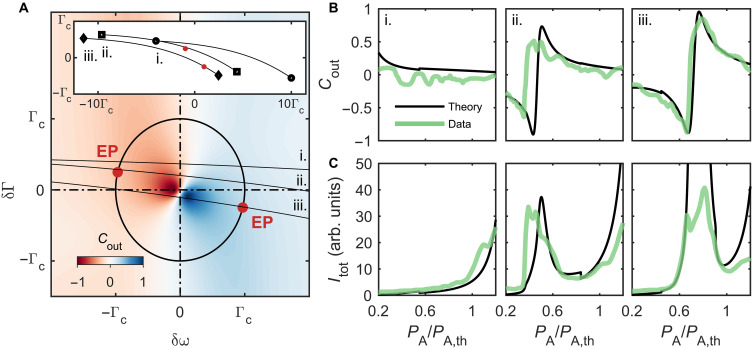
Directionality switching near EPs. (**A**) Theoretical output contrast calculated in the parameter space {δω, δΓ} with reported positions of the EPs (red dots). Here, we assume balanced optical pumping powers. Three trajectories (i., ii., and iii.) are computed on the basis of the experimental calibrations and indicate the path associated to the experimental measurements shown in (B) and (C). The full trajectories are reported in the inset. (**B**) Output contrast and (**C**) total output intensity measured as a function of the pump power *P*_A_. The measurement (green data) is reproduced at different values of the heating current and of the pump power *P*_B_. Theoretical curves are computed based on the nanolaser calibrations (black curves).

In Fig. 4A, we plot the theoretical output contrast in the parameter space {δω, δΓ}, inputting the calibrated experimental parameters in the model. Both EPs (red dots) are represented on the circle $\delta \omega^{2}+\delta \Gamma^{2}=\Gamma_{c}^{2}$. The colormap evidences an abrupt transition from left-side emission (blue lobe) to right-side emission (red lobe) regions. The transition between the two EPs corresponds to the path that offers the most contrasted change of directionality. Relying on experimental calibration, we convert the pump powers and the heating current into a position of the system in the parameter space {δω, δΓ}. In particular, in Fig. 4A, we show three different trajectories obtained by sweeping the pump power *P*_A_, while *P*_B_ still remains constant. These trajectories are dominated by a frequency detuning contribution, which reflects the Henry factor (*34*) that we have calibrated, α_H_ ≈ 20. By varying *P*_B_ and *i*_th_, we explore various trajectories and observe different types of responses.

The measured output contrast associated to each curve is shown in Fig. 4B, accompanied by the total output intensity in (C). The trajectories—labeled i., ii., and iii.—get progressively close to the point {0,0}, where the loss splitting reaches its maximum. Whereas the response in i. is essentially given by the single nanolaser (A) response, a collective mode builds up in ii. when the nanolaser frequencies cross each other. In iii., the hybridization is maximum, leading to a substantial increase on the QNM response around *P*_A_/*P*_A,*t*_ = 0.4. Moreover, the output contrast experiences an abrupt inversion, switching from −0.87 to +0.85 in an interval of only 9% of the threshold power *P*_A,*t*_.

The theoretical data shown in Fig. 4A are computed assuming balanced pumping (δ*P* = *P*_A_ *− P*_B_ = 0), which is useful to represent the general behavior of the output contrast in {δω, δΓ}. However, this model does not completely match the experimental conditions where one of the pump powers is swept, while the other is fixed, i.e., with a significantly varying δ*P*. To enable a quantitative modeling of the experiments, the calibrated nanolaser parameters are injected into the coupled mode theory model from which the theoretical output contrast and total output intensities are computed as shown with black curves in Fig. 4 (B and C). The theory captures well the observations, and the small discrepancies can be imputed to some drifts in the experiment, e.g., of the pump lasers alignments or of the temperature in the heating nanowires. The coupled mode analysis does not account for the nanolaser nonlinear saturation such that negative decay rate translates into an infinite intensity as observed in Fig. 4Ciii.

## DISCUSSION

We demonstrated that the usual frequency avoided crossing observed when detuning two coupled resonators turns into a strong loss splitting if the phase shift is set to a multiple of π. The resulting collective lasing emission is characterized by a spatial directionality that can switch depending on the position of the system relative to the EPs, in differential parametric space, {δω, δΓ}. While the emission is perfectly balanced (*C*_out_ = 0) when ϕ = 0 ( mod π), as directly shown by Eq. 3, the directionality switching is particularly abrupt in the immediate neighborhood of these points. Consequently, these are phase singularities that motivate the implementation of a direct modulation of the phase shift between the nanolasers for their practical utilization (*35*, *36*).

The directionality switching bandwidth has yet to be determined but is expected to be limited by the photon lifetime, likely in the range of tens of gigahertz. In the future, the use of electrical injections (*37*) would offer an ultimately integrated device in which the directionality could be fully remotely determined. The association of directionality with nonlinear dynamical regimes could be exploited for the realization of all-optical memories (*38*). Similarly, self-pulsing or excitable regime encountered in the vicinity of EPs would certainly be associated with the spatial nonreciprocity detailed here (*39*). More generally, the present platform is an ideal candidate for the exploration of novel laser dynamics occurring in the vicinity of EPs (*40*–*42*) and for the join study of nonlinear non-Hermitian photonics.

## MATERIALS AND METHODS

### Sample fabrication process

We use a silicon-on-insulator (SOI) chip containing 2.5-mm-long ridge waveguides with varying widths enabling different coupling strength with the active cavities (*29*). The 280-nm-thick InP-based layer is adhesively bonded with benzocyclobutene on top of the SOI circuit. The nanolasers are defined using e-beam lithography (EBL) on hydrogen silsesquioxane and patterned in the III-V layer with inductively coupled plasma etching (ICP). Chemical surface passivation based on ammonium sulfide (*29*) is used to remove non-radiative carrier recombination defects. A 1-μm-thick layer of silica is deposited with plasma-enhanced chemical vapor deposition to encapsulate the nanolasers. Last, metallic nanowires to be used as heaters are made above the nanolasers using EBL, Ti/Au evaporation (10/60 nm), and liftoff.

### Derivation of the output directionality

We develop the coupled mode analysis used to compute the waveguide output intensities and deduce the output contrast. We start from the rate equations describing two waveguide-coupled nanolasers

$$i\frac{dA}{\mathrm{dt}}=\left(\begin{array}{cc} \omega_{A}-i\Gamma_{A} & -iK\\ -iK & \omega_{B}-i\Gamma_{B} \end{array}\right)A+\left(\begin{array}{c} s_{A}\\ s_{B} \end{array}\right)$$ (4)

where *s*_A_ and *s*_B_ are source terms that translate the spontaneous emission in the cavities. We deduce the stationary solutions by imposing *a*_A,B_ = *r*_A,B_*e*^*i*ω*t*^ and solving Eq. 4

$$r_{A}=\frac{\left(\omega -\omega_{B}+i\Gamma_{B}\right)s_{A}+Ks_{B}}{\left(\omega -\omega_{A}+i\Gamma_{A}\right)\left(\omega -\omega_{B}+i\Gamma_{B}\right)-K^{2}}$$ (5)

$$r_{B}=\frac{\left(\omega -\omega_{A}+i\Gamma_{A}\right)s_{B}+Ks_{A}}{\left(\omega -\omega_{A}+i\Gamma_{A}\right)\left(\omega -\omega_{B}+i\Gamma_{B}\right)-K^{2}}$$ (6)

From which we compute the total output intensity and the output contrast, with in mind that *s*_L_ = *r*_A_ + *e*^*i*ϕ^*r*_B_, and *s*_R_ = *e*^*i*ϕ^*r*_A_ + *r*_B_. We compute *C*_out_ assuming a linear growth of photon number below threshold, i.e., $s_{A,B}\propto \frac{P_{A,B}}{P_{A,B,t}}$. Here, *P*_A,B_ is deduced from Γ_A,B_, through the calibration functions. The mean loss rate, $\bar{\Gamma }$, is taken constant and equal to Γ_c_/2 in the colormap shown in Fig. 4A.

### Pump power dependence of the total dissipation rate

The amplitude decay rate Γ*_k_* of cavity *k* can be split into three components

$$\Gamma_{k}=\Gamma_{\text{in}}+\Gamma_{c}-\Gamma_{g,k}$$ (7)

where Γ_in_ is the cavity intrinsic decay rate. Γ_*g*,*k*_ is a gain term—this is why it is assigned to a minus sign—that depends on the carrier density, *N_k_*, in the semiconductor and, by extension, to the optical power of the pump. We write

$$\Gamma_{g,k}\left(N_{k}\right)=\frac{V_{a}}{2}G\left(N_{k}\right)\;\text{where}\;G\left(N_{k}\right)=G_{0}\;\text{ln}\left(\frac{N_{k}+N_{s}}{N_{\text{tr}}+N_{s}}\right)$$ (8)

Here, *G*_0_ is a gain factor that depends on the materials and cavity properties, *N*_tr_ is the carrier density at transparency, and *N_s_* is a residual carrier density. The nanolaser wavelength is also affected by the carrier population that fills the electronic levels in the semiconductor, leading to a blueshift of the cavity.

Below threshold, we assume a linear dependence of the carrier density with the pump power: *N_k_* = ε*P*, before a clamping of the carrier density above threshold. Overall, the amplitude decay rate of cavity *k* reads

$$\Gamma k\;\left(P\right)=\Gamma_{0}+\Gamma_{c}-g\;\text{with}\;\Gamma_{0}=\Gamma_{\text{in}}+\frac{V_{a}}{2}G_{0}\;\text{ln}\left(\frac{N_{s}}{N_{S}+N_{\text{tr}}}\right)\;\text{and}\;g=\frac{V_{a}}{2}G_{0}\;\text{ln}\left(\varepsilon \frac{P}{N_{s}}+1\right)$$ (9)
