# Supplementary material for: Harnessing coupled nanolasers near exceptional points for directional emission
Source: Sci Adv. 2024 Nov 8;10(45):eadr8283. doi: 10.1126/sciadv.adr8283 (PMC11546807; doi:10.1126/sciadv.adr8283)
Supplement: Supplementary file 1 — Supplementary Text Figs. S1 to S7 Table S1 [file sciadv.adr8283_sm.pdf]

Science Advances
AAAS

Supplementary Materials for

Harnessing coupled nanolasers near exceptional points for directional emission

Guilhem Madiot *et al.*

Corresponding author: Guilhem Madiot, gmadiot@unice.fr

Sci. Adv. **10**, eadr8283 (2024)
DOI: 10.1126/sciadv.adr8283

This PDF file includes:

Supplementary Text
Figs. S1 to S7
Table S1

Calibration of the thermoresistive nanowires

In this section, we detail how the thermoresistive nanowires are used and calibrated. The physical mechanism corresponds to a thermo-optic shift of the nanolaser cavities. A current i_{th} is injected into the nanowire by contacting both electrodes with Tungsten microtips. To calibrate the heating constant, β_{th}, we measure the output intensity spectrum while increasing the heating current. The red-shift experienced by the nanocavity under heating of its constitutive material (InP) is proportional to the absorbed power, i.e. to $P_{\mathrm{th}} = R_{\mathrm{th}} i_{\mathrm{th}}^2$, with R_{th} the nanowire resistance. R_{th} is typically of the order of 50 Ω. We pump a single nanolaser above its lasing threshold such that its emission wavelength can be unambiguously determined. Importantly, the other nanolaser is not pumped, such that its resonance wavelengths are far detuned from the cavity of interest. Indeed, one must ensure there is no coupling between the cavities during this operation, to certify that the observed spectrum corresponds only to the heated cavity.

In Fig. S1, we plot the emission wavelength (A) as a function of the squared input current i_{th}^2. We fit the data to a linear function and obtained $\beta_{\mathrm{th}} = 1.94$ nm/µW. The linewidth is limited by the spectroscope resolution in this regime, but a close look at the emission intensity (B) shows that the temperature also slightly affects the laser linewidth. Most likely here, the effect observed in Fig. S1 B arises from a spectral shift of the gain zone with regard to the cavity resonance wavelength. This effect is neglected in the data analysis.

Fitting of the eigenspectrum

The experimental spectra are measured using an IR spectrometer with a spectral resolution $\sigma \approx 0.2$ nm. Below the laser threshold, the nanolaser emission lineshape follows a Lorentzian distribution. In the case of coupled nanolasers, the output spectrum consists of a sum of two Lorentzian modes:

$$s_{out} = \frac{A_-}{(\lambda-\lambda_-)+\Delta\lambda_-} + \frac{A_+ e^{j\theta}}{(\lambda-\lambda_+)+\Delta\lambda_+} + B$$

With $A_\pm$, $\lambda_\pm$, $\Delta\lambda_\pm$, θ,and B real quantities. $\lambda_\pm$ and $\Delta\lambda_\pm$ are the eigenmodes resonance wavelengths and full-widths at half-maximum, respectively. $A_\pm$ represents their respective amplitudes and θ describes an interference between the two modes, enabling Fano-like lineshapes. B is the noise floor. The output intensity reads $I = |s_{out}|^2$. Near and above threshold, the emitting collective modes have a linewidth below the spectrometer resolution, such that the observed spectrum is rather a convolution of a Lorentzian lineshape with a Gaussian distribution, i.e. a Voigt lineshape. Therefore, we fit each spectrum with a sum of two dephased Voigt lineshapes. In Fig. S2, we show three typical situations encountered in the data analysis. **A**: the Quasi-Normal Modes (QNMs) have both frequency- and loss-splittings. The low-wavelength mode is broader and less intense, while the high-wavelength mode is narrower and more intense. The second is actually narrower than the spectral resolution, so its lineshape looks more Gaussian than Lorentzian, which is well captured by the Voigt fit (solid line). **B.** The QNMs have identical resonance wavelengths, but different linewidths, with one (low-intensity) broad resonances on top of which a narrow resonance sits. The latter is lasing, and its linewidth is limited by the spectroscope resolution. **C**. The QNMs have identical linewidths but separate spectral positions. The peaks have a Lorentzian profile and show similar intensities.

Nanolaser calibration

The nanolaser calibration is carried out by measuring the output intensity spectrum while increasing the associated pump power, P. The spectra are fitted using a Voigt function with a Gaussian width set by the spectrometer resolution to 0.1 nm. In Fig. S3 A, we show the power-dependent spectra of nanolaser A as used in Figs.3-4 of the manuscript. The nanolaser intensity (I), wavelength (λ_A), and linewidth (Full-Width Half-Maximum: FWHM), $\Delta\lambda_A$, are plotted in Fig S3 B, C, and D, respectively.
Relying on Eq. (9) in the manuscript, we model the wavelength and FWHM of a single laser below threshold with a function of the form:

$$f(P) = y_0 - A \ln\left(\frac{P}{B} + 1\right)$$

where y_0, A, and B are constants to determine. The fitted experimental data are shown with the red line in Fig. S3 C-D. We deduce the Henry factor, α_{H}, from the mean ratio between the wavelength shift and the linewidth shift. We calibrate the nanolaser B with the same methodology and report all calibration quantities in Table S1. The values obtained for the two nanolasers are consistent with each other. After this procedure, it is possible to determine the linewidth and wavelength of the calibrated nanolaser by using the reverse functions for any applied power below threshold. In Figures 4 of the manuscript, any set of power $\{P_A, P_B\}$ applied to the system can be converted into the parameters $\{\omega_A, \omega_B, \Gamma_A, \Gamma_B\}$, which directly provides a unique position in $\{\delta\omega, \delta\Gamma\}$.

Calibration of the internal and external decay rates

We can access the internal decay rate Γ_{in} and the external decay rate Γ_c by fitting the normalized transmission spectrum of the waveguide with a Lorentzian dip, provided that the cavities are unpumped and made independent from each other, e.g. by red-shifting the undesired cavity with the heating nanowire. The transmission function reads

$$T(\lambda) = (\Delta^2 + \Gamma_{in}^2)/(\Delta^2 + (\Gamma_{in} + \Gamma_c)^2),$$

where $\Delta = 2\pi c\left(\frac{1}{\lambda} - \frac{1}{\lambda_0}\right)$. λ_0, Γ_{in} and Γ_c are the fitting parameters. In Fig.S4, we show a typical measurement (blue dots) produced on the structure on which the data shown in Figs.3-4 of the manuscript are obtained. The waveguide is injected with a Super-Luminescent Diode (SLD) in the near-infrared and the output spectrum is normalized and fitted with the above expression (red line). We obtain an internal decay rate $\Gamma_{in} = 27.2 \pm 4.1$ GHz and an external decay rate $\Gamma_c = 22.6 \pm 1.6$ GHz.

Lasing curve of the collective mode

We can detail the loss contributions of cavity k as follows

$$\Gamma_k = \Gamma_0 + \Gamma_c - g_k$$

where Γ_0 accounts for both the internal loss rate and the absorption rate of the unpumped cavity, Γ_c the external loss rate, and g_k the gain term applied to cavity k (see Eq. 7 of the manuscript). Assuming that the cavities are tuned ($\omega_A = \omega_B$) and symmetrically pumped ($g_A = g_B \equiv g$), then the coupling between cavities A and B through a coupling phase $\phi = 0$ or $\phi \approx \pi$ leads to a broadening of one eigenmode ($\Gamma_- = \Gamma_0 + 2\Gamma_c - g$) and a narrowing of the other mode ($\Gamma_+ = \Gamma_0 -$

g) such that the lasing threshold of the former decreased by a significant amount compared to the single-nanolaser situation.[1] If the cavity is pumped below threshold but such that the internal losses are compensated by the gain ($\Gamma_0 < g < \Gamma_0 + \Gamma_c$), then the collective mode (+) can lase.
To verify that the collective mode considered in Figure 4 is lasing, we perform a measurement of the collective mode lasing curve on the structure used in Fig.3 and 4 of the manuscript. After setting the frequency detuning $\delta\omega$ to zero, we ramp up identical pump powers onto the cavities ($P_A/P_{A,t} = P_B/P_{B,t}$) and measure the spectrum. In Fig.S5, we show the peak position (A), linewidth (B), and intensity (C) as a function of the normalized pump power.
The lasing threshold is found around $P_k/P_{k,t} \approx 0.5$ and manifests by the sudden increase in the emission intensity as well as the clamping of both the emission wavelength and the emission linewidth. Note that the ratio between the intrinsic and external losses in the cavity can be deduced from this value:

$$r = \frac{\text{threshold of collective mode}}{\text{threshold of single cavity}} \approx 0.5.$$

Meanwhile, from the relations detailed above in this section, we can express $r = \Gamma_{in}/(\Gamma_0 + \Gamma_c)$. From the independent measurement shown in Fig.S4, we obtain $r \approx 0.55 \pm 0.15$.

To conclude, the pump power used in the experiment in Fig.3 of the manuscript is at $P_k/P_{k,t} \approx 0.65$ when the directionality flips, which is above the collective mode lasing threshold. In Fig.4, the pump ramp is performed below the collective mode lasing threshold (*i.*), near the threshold ($P_k/P_{k,t} \approx 0.5$, in *ii.*), and above the threshold ($P_k/P_{k,t} \approx 0.75$, in *iii.*). Note that the comparison between the data in Fig.S4 and the data in Fig.3 is only possible when the normalized pump powers are equal, i.e. in the diagonal of the colormap in Fig.3 A-B. This corresponds to the zero-contrast point of the data shown in Fig.3 D-E.

T heoretical influence of ϕ and Γ_0 on the output contrast

We numerically compute the output contrast in the parameter range $\{\delta\omega, \delta\Gamma\}$, for varying phase-shifts (Fig. S5), and for varying internal loss-rates (Fig. S6). In Fig. S5, we use the set of numerical parameters $\frac{\overline{\omega}}{2\pi} = 1.8855$ THz, $\Gamma_c/(2\pi) = 20$ GHz, $\Gamma_0 = \Gamma_c$, and $s_a = s_b$, which is very close to the experimental parameters calibrated in the manuscript. We note the similarity of the case $\phi = 1.1\pi$ with the theoretical plot in Fig. 4 A of the manuscript, where $\phi = 1.08\pi$. When tuning the phase shift away from π, the output contrast tends to change less abruptly when crossing the point $\{0{,}0\}$. Moreover, one should keep in mind that slightly moving towards $\phi = \pi/2[\pi]$ implies the two eigenmodes tend to have identical linewidths and separate frequencies. Thus, at $\phi = \pi/2$, two modes coexist with exact same losses, and separated by $2\Gamma_c$ in the spectrum. In the numerics, we pick the lowest loss-rate QNM, which is an arbitrary choice based on the fact that the experiment is realized with a pair of cavities set in a strong Q-splitting regime, i.e. where the low-Q mode does not significantly contribute to the observed emission. This is not true near $\phi = \pi/2$, where the output spectrum is strongly multimode.

At $\phi = \pi$, as expected from a simple replacement in Eq.3 of the manuscript, the output contrast is zero. This is due to the fact that the low-loss mode has external losses that reach exactly zero, i.e.

[1] The notation +/- for the eigenmodes is arbitrarily chosen here.

it becomes dark. Yet, at this specific point the mode is conditioned to maximize its internal photon number, especially if the net loss rate becomes negative, which indicates that the mode passes its lasing threshold. Therefore, the intensity and the output contrast strongly vary in the immediate neighborhood of this particular phase, and $\phi = 0[\pi]$ is a phase-singularity.

Now, using a constant phase-shift $\phi = 1.1\pi$ and tuning the internal loss rate Γ_0 shows how the abruptness of the directionality flip is set by the decay rate ratio $\gamma = (\Gamma_0 - g)/\Gamma_c$. In Fig. S6, we show the same colormap with varying values of γ, from negative values (the lasers are pumped above their threshold) to positive values (the lasers are pumped below threshold, or unpumped). Note that the scale differs from Fig. S5 in order to show the entire evolution of C_{out} for high values of γ. The plot evidences how the abruptness of the distance from a minimum to a maximum of the output contrast is set by γ.

Article I.

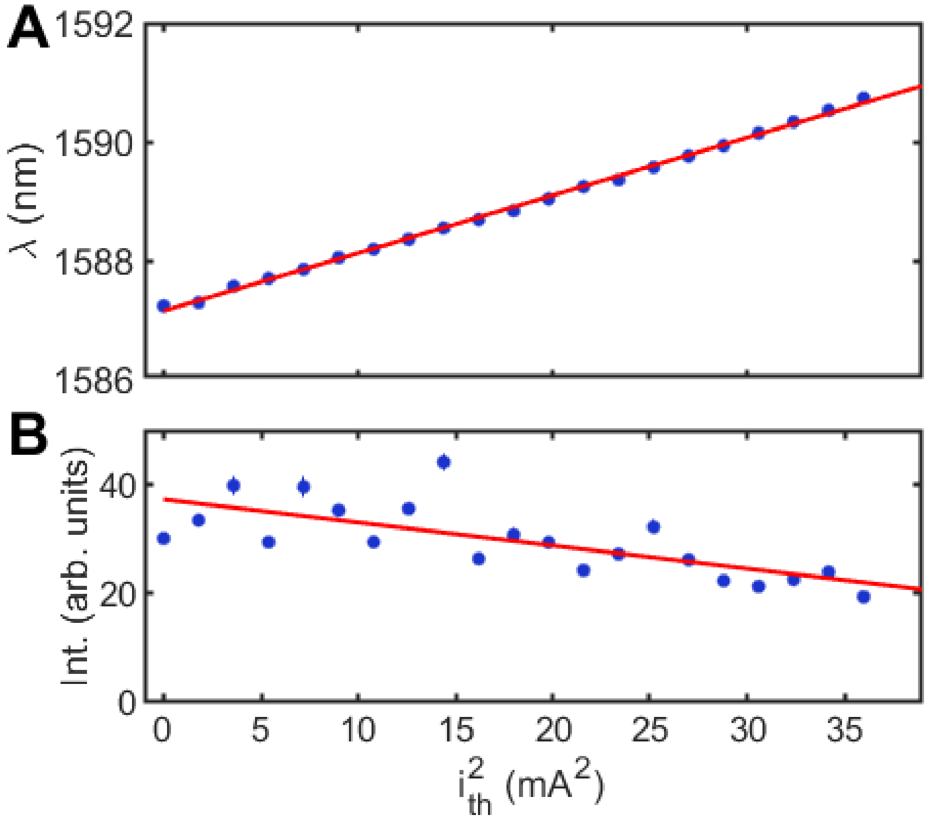

Fig. S1. Calibration of the thermoresistive nanowire. A nanolaser emission wavelength obtained by fitting the output spectra, as a function of the squared input current. The data (blue dots) are fitted with a linear function (red line) which returns the calibrated heating constant $\boldsymbol{\beta}_{\mathbf{th}}$=1.94 nm/µW. **B** The nanolaser emission intensity slightly decreases with the temperature. The data (blue dots) are fitted with a linear function (red line).

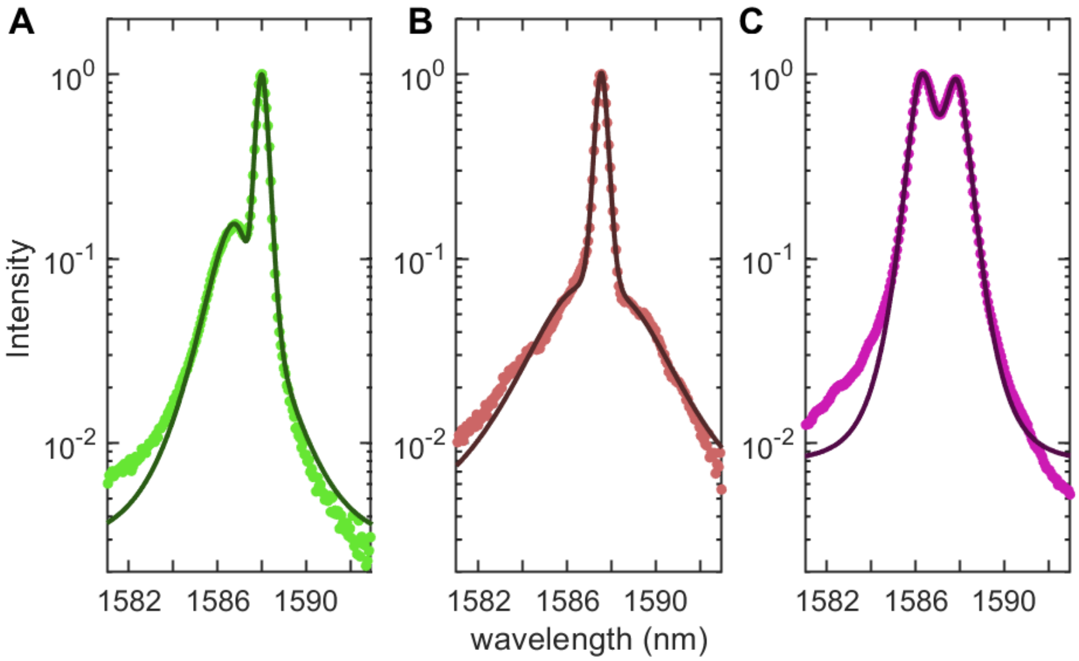

Fig. S2. Double-Voigt fitting of the collective modes. Experimental spectra measured in different structures (dots), fitted with a sum of two interfering Voigt-shape amplitudes. We show three examples illustrating the typical cases (**A**) complex splitting with both energy repulsion and Q-splitting **(B),** pure Q-splitting, and **(C)** pure energy-splitting. Note the logarithmic y-scale.

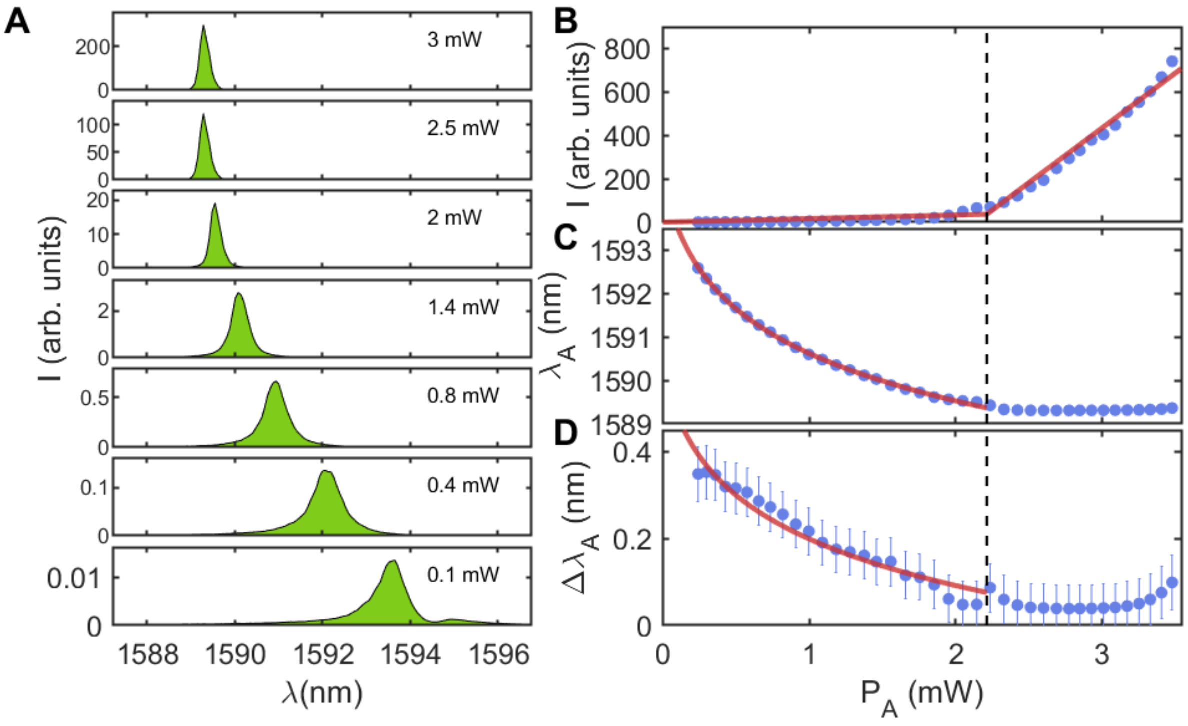

Fig. S3. Calibration of nanolaser A (**A**) Some output intensity spectra are shown for various pump powers. The spectra are fitted with a Voigt function to extract (**B**) the measured emission intensity that we fit with a threshold function (red line) which returns the nanolaser threshold power $\boldsymbol{P_{A,t} = 3.97}$ mW (black dashed lines). (**C**) the laser wavelength, $\boldsymbol{\lambda_A}$ is blue-shifted below threshold; and (**D**) The laser linewidth, $\boldsymbol{\Delta\lambda_A}$, reduces down to the spectroscope resolution, and remains constant above the laser threshold. The laser intensity is fitted with a threshold function. Both the laser wavelength and linewidth are fitted with logarithmic function that model the gain function below threshold (red lines).

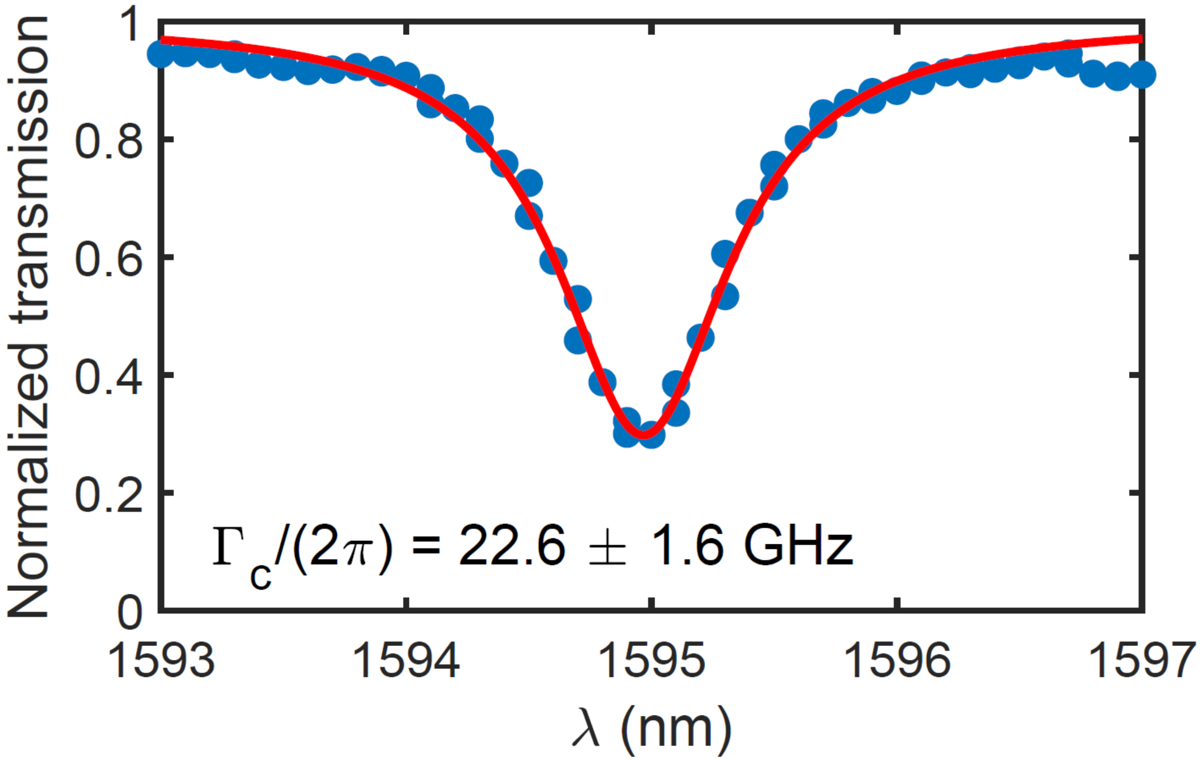

Fig. S4. Calibration of Γ_c Measured output spectrum of the integrated waveguide injected with a broadband SLD. The dip corresponds to the unpumped cavity B, while the cavity A is red-shifted using the associated heater.

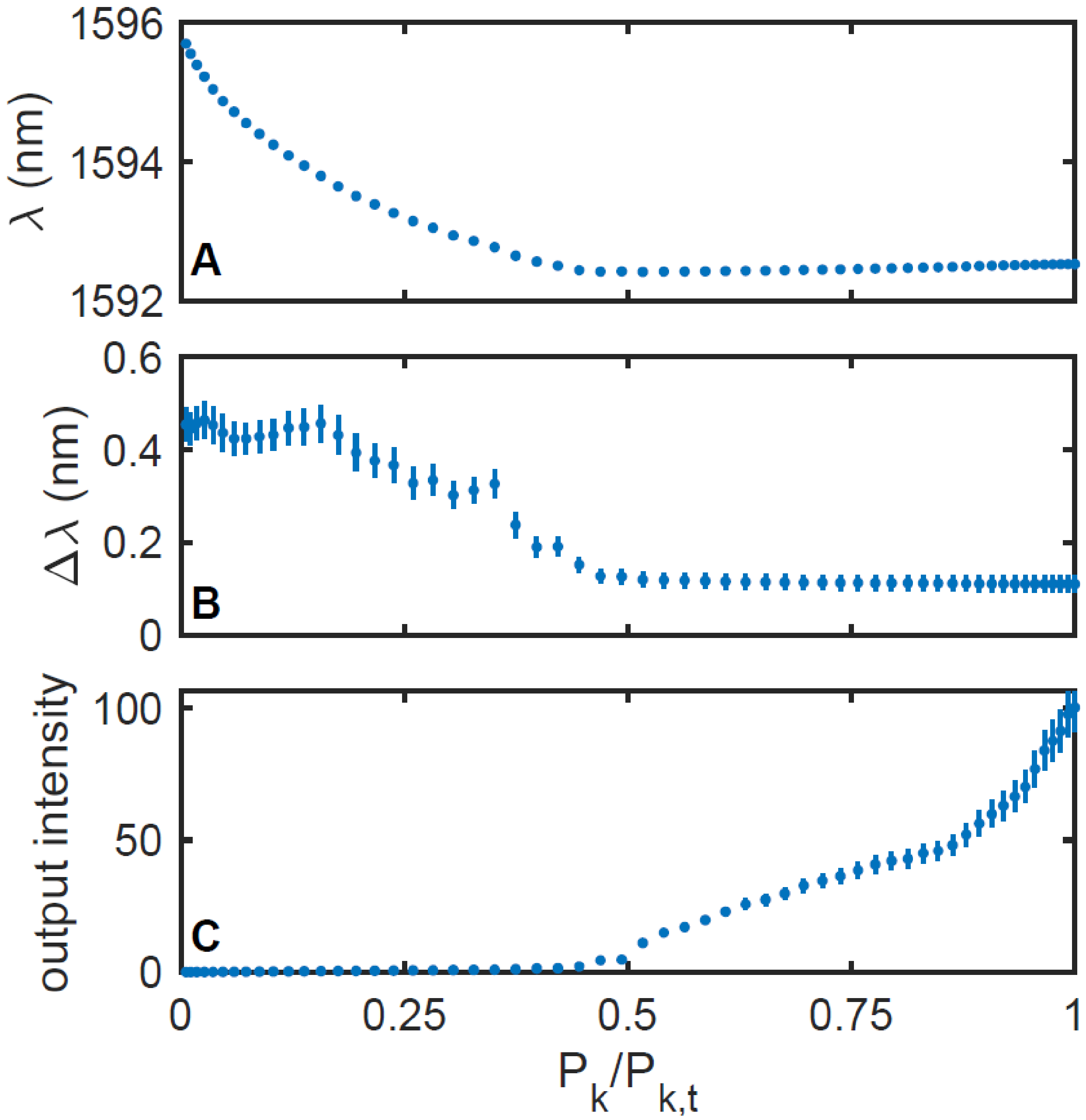

Fig. S5. Phase dependence of the output contrast Measured emission wavelength (A), linewidth (B), and intensity (C) as a function of the normalized pump power $\boldsymbol{P_k/P_{k,t}}$ applied simultaneously to both cavities ($\boldsymbol{P_A/P_{A,t} = P_B/P_{B,t}}$). The nanolasers are coupled via a phase $\boldsymbol{\phi \approx 1.08\pi}$. This measurement is performed on the structure used in Fig.3-4 or the manuscript.

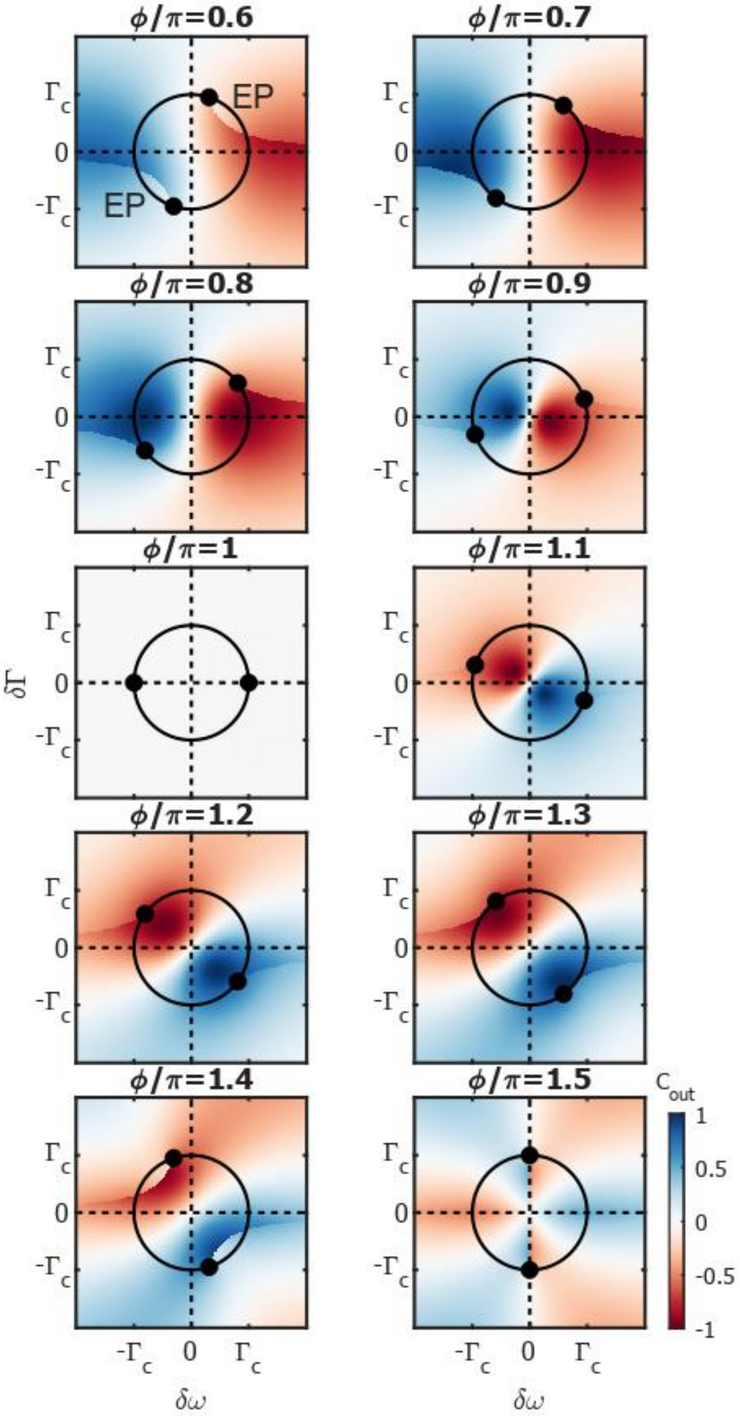

Fig. S6. Phase dependence of the output contrast

Theoretical output contrast in the parameter space $\{\delta\omega, \delta\Gamma\}$, for varying phase-shift. We use $\frac{\overline{\omega}}{2\pi} = 1.8855$ THz, $\Gamma_c/(2\pi) = 20$ GHz, $\gamma = 1$, and $s_a = s_b$. The exceptional points (EP) are shown with black dots. We highlight the circle $\delta\omega^2 + \delta\Gamma^2 = \Gamma_c^2$.

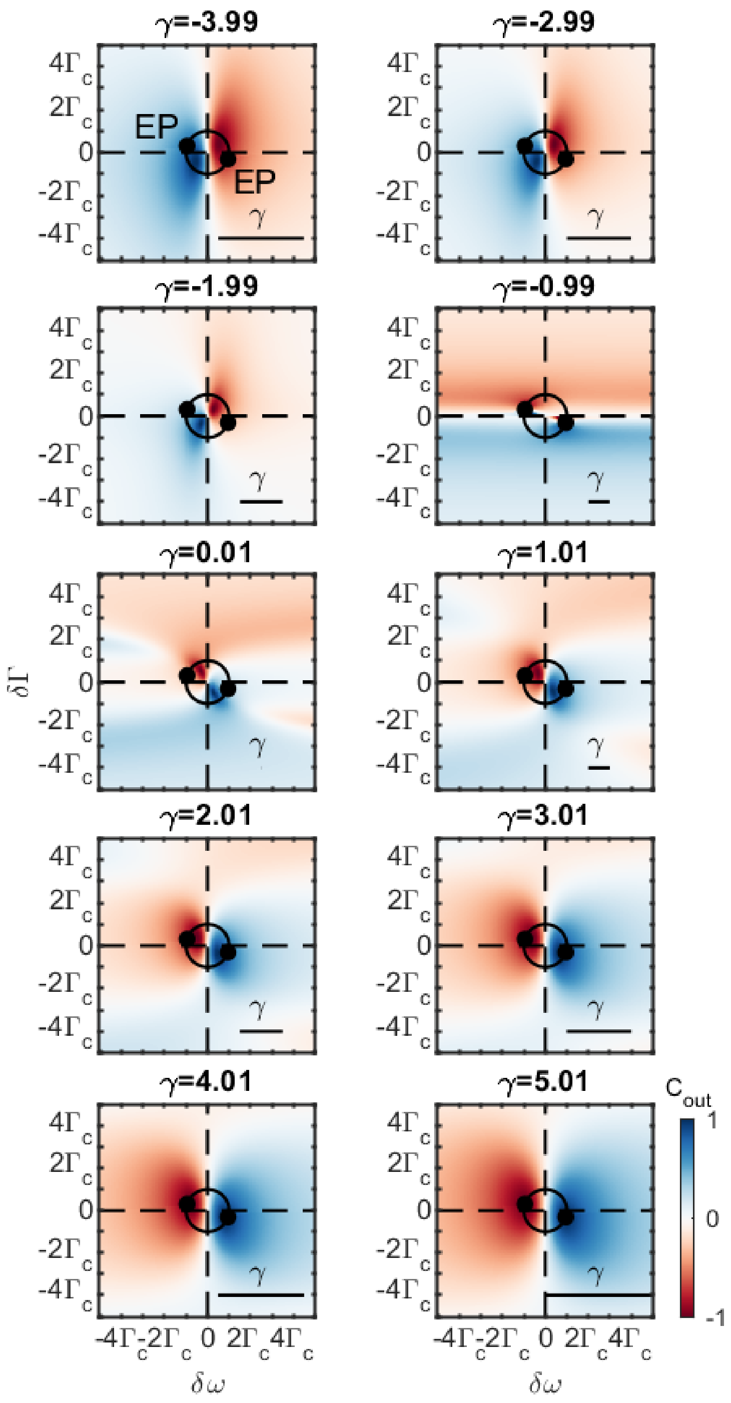

Fig. S7. Phase dependence of the output contrast

Theoretical output contrast in the parameter space $\{\delta\omega, \delta\Gamma\}$, for varying ratio decay rate $\gamma = (\Gamma_0 - g)/\Gamma_c$. We use $\frac{\overline{\omega}}{2\pi} = 1.8855$ THz, $\Gamma_c/(2\pi) = 20$ GHz, $\phi = 1.1\pi$, and $s_a = s_b$. The exceptional points (EP) are shown with black dots. We highlight the circle $\delta\omega^2 + \delta\Gamma^2 = \Gamma_c^2$. We show the scale Γ_0/Γ_c with a black bar to indicate the typical evolution scale of the output contrast.

Quantity (units)	Nanolaser A	Nanolaser B
β_{th} (nm/mW)	1.94$\pm$0.07	-
P_t (mW)	2.2$\pm$0.2	4.3$\pm$0.2
λ_0 (nm)	1594.64	1594.73
$\Delta\lambda_0$ (pm)	620$\pm$110	492$\pm$70
$\Gamma_c/(2\pi)$	19.5$\pm$4.6	22.6$\pm$1.6
α_{H} (none)	19.8$\pm$5	26.8$\pm$5

Table S1.
Calibration parameters of the pair of nanolasers used in Figs.3-4 of the manuscript.
